# Low-cost photodetector architectures fabricated at room-temperature using nano-engineered silicon wafer and sol-gel TiO_2_ – based heterostructures

**DOI:** 10.1038/s41598-019-54481-8

**Published:** 2019-11-29

**Authors:** Debika Banerjee, Ivy M. Asuo, Alain Pignolet, Sylvain G. Cloutier

**Affiliations:** 1 1Dept. of Electrical Engineering, École de Technologie Supérieure, 1100 Notre-Dame Ouest, Montréal, QC H3C 1K3 Canada; 2 Institut National de la Recherche Scientifique (INRS), 1650 Boul. Lionel Boulet, Varennes (QC), J3X 1S2 Canada

**Keywords:** Electronic devices, Optoelectronic devices and components, Electronic devices

## Abstract

In the last decades, significant research has been done on the nanocrystalline forms of titanium dioxide (TiO_2_). Amorphous TiO_2_ has not been studied intensively despite being significantly less expensive compared to crystalline TiO_2_. This study reveals significant improvement in UV-VIS photodetection properties from heterostructures fabricated in ambient environment using n-type silicon nanowire arrays and amorphous TiO_2_ sol-gel. Our ultra-low-cost UV-VIS photodetectors can cover a wide range of applications. We report fast rise/decay time constants of 0.23 ms/0.17 ms and high responsivity up-to 6.0 A/W in the UV and 25.0 A/W in the visible range under low (1 V) external bias. The large surface area due to the nanowire array architecture leads to 2 orders of magnitude enhancement in photo-response. Besides the final electrode deposition, the entire device fabrication is performed using low-cost, all solution-based methods in ambient conditions. These low-cost UV-Visible broadband photodetectors can potentially serve a wide range of applications.

## Introduction

Silicon nanowires (SiNWs)-based hybrid heterojunction have generated tremendous interest over the last decade for their potential use in photo-catalytic, energy-harvesting and sensing devices^1–3^. TiO_2_-based photodetectors are already widely used for UV detection^4–6^. Significant efforts were made to fabricate low-cost photodetectors serving various applications by fabricating p-n junction, heterojunction or Schottky junction for their excellent charge-separation ability^7–9^. Although TiO_2_-based photodetectors are attractive for their UV detection ability, they suffer from low absorption and hence low responsivity due to their large band gap^10^. It was already proposed to improve the photo-responsivity of TiO_2_-based photodetectors by forming a heterojunction with other narrow bandgap materials like Si to extend its detectivity to the visible^11,12^. Hence, fabricating heterojunction using silicon and TiO_2_ can help absorbing light across the UV and the visible^13^. However, most efforts have focused on improving the detection properties using crystalline TiO_2_^14^. Nevertheless, amorphous TiO_2_ can also provide a larger surface area and enhanced absorptivity, but at much lower processing costs^14^. Using nanostructured materials can significantly improve the performances of such heterojunction-based devices^15–18^. Indeed, TiO_2_ nanostructuring has been previously used to boost responsivity from Si-TiO_2_ photodetectors^13^. Crystalline TiO_2_ nanorods have also been successfully used to similar ends^19,20^.

In this paper, we have explored the promising performances of simple SiNWs/TiO_2_ heterojunction devices produced using a facile all-solution based approach. To do so, we fabricate a dense vertically-aligned SiNWs array by applying all solution-based galvanic displacement chemistry to a commercial n-type silicon wafer. Thereafter, amorphous and anatase crystalline TiO_2_ have been deposited for comparison. We observe excellent photodetection properties from our devices compared to the state-of-the-art^21,22^. While our results suggest that using amorphous TiO_2_ proves slightly less efficient compared to anatase TiO_2_, it certainly provides a much energy-efficient and straightforward process alternative by avoiding the high-temperature conversion from amorphous to anatase TiO_2_.

## Experimental Methods

### Fabrication of the vertically-aligned silicon nanowire arrays

**Figure 1:**
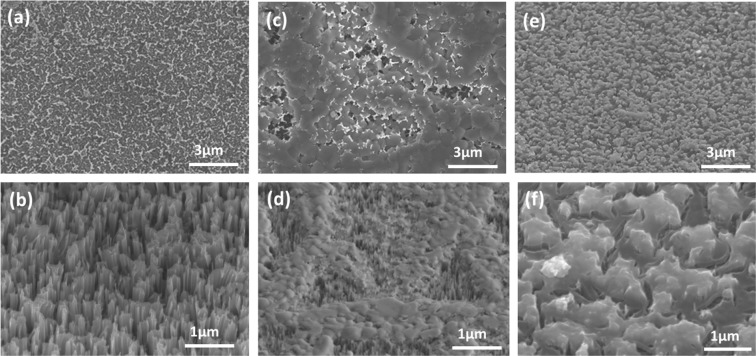
(**a**) Top-view and (**b**) 45° tilted-view of the vertically-aligned silicon nanowire arrays under SEM. (**c**) Top-view and (**d**) 45° tilted-view of the nanowire arrays after amorphous TiO_2_ deposition. (**e**) Top-view and (**f**) 45° tilted-view of the nanowire arrays after anatase TiO_2_ deposition.

Silicon nanowire arrays are fabricated using immersion-based galvanic displacement (GDM) chemistry directly on a commercial n-type silicon wafer. The complete fabrication process is already fully-described elsewhere^1,2,23^. Here, we use an n-type phosphorous doped silicon wafer with 1–10 ohm.cm resistivity purchased from UniversityWafers. The unit Ohm-cm indicates the bulk resistivity of the silicon wafer. For the etching solution, 0.02 M silver nitrate (AgNO_3_) and 5 M hydrogen fluoride (HF) aqueous solutions are mixed in 1:1 ratio to prepare the etchant. Silicon pieces are cleaved into 4 cm^2^ pieces using a carbide-tip pen. Then the silicon pieces are cleaned by ultra-sonication in acetone and isopropyl alcohol for 10 minutes each. Finally, the samples are washed with deionized (DI) water and dried with a nitrogen gun. The cleaned samples are transferred to the etching solution immediately. The immersion lasts 40 minutes at room temperature. After etching, the resulting vertically-aligned nanowire arrays are covered by silver dendrite layer^16^. This residual silver is entirely removed using 70% nitric acid (HNO_3_) for 1 hour at room temperature^16^. Figure 1(a,b) show the top-view and 45° tilted-view SEM micrographs of the final silicon nanowire arrays. The nanowires seen in Fig. 1(a,b) are between 800–1000 nm in length, and their diameters range between 40–60 nm.

### Deposition of amorphous and anatase TiO_2_ layer

**Figure 2:**
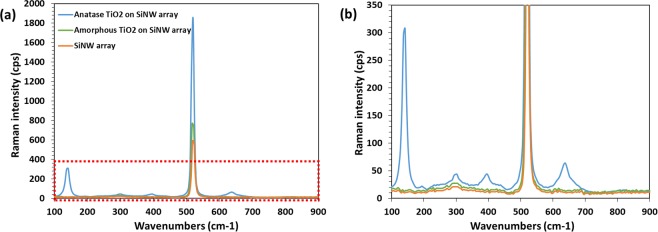
(**a**) Raman micro-spectroscopy results to confirm the pristine SiNWs, amorphous TiO_2_ atop the nanowires and anatase TiO_2_ phase atop the nanowire arrays. (**b**) Zoom in part of the selected area (red box) of the spectra from (**a**).

The SiNWs samples are treated with 20% hydrobromic acid (HBr) solution for 2 minutes for the removal of the native oxide layer from the top of the nanowires. Few nanometers of native oxide grow naturally on any bare Si surface under standard atmospheric conditions. Generally, this native oxide layer is removed by treating the Si surface with 2% HF solution^24^. We have evaluated the effects of different surface treatments by performing contact angle measurements. 10 µl of TiO_2_ droplet has released on each of the non-treated, 2% HF-treated and 20% HBr-treated SiNWs samples using a micro syringe. The TiO_2_ droplet images are taken from the side view of the sample with a high-resolution camera. The contact angle is estimated by image analysis in image J software employing low bond axisymmetric drop shape analysis (LBADSA) plugin^25,26^. The LBADSA plugin is attributed to the fitting of the image data with the Young-Laplace equation. The schematic of the droplet on the nanowire sample has shown in Fig. S1 in the supplementary information section. We have observed 45.6°, 39.7° and 25.3° contact angles for non-treated, HF-treated and HBr-treated devices respectively. As we know, the higher the contact angle means lower the wettability of the TiO_2_ sol-gel solution onto the nanowire substrates, HBr-treated devices have better wettability of TiO_2_ than the other two devices. A poorer wetting could leave more voids between the nanowires underneath the TiO_2_ film, which in turn affects the performance of the heterojunction. Hence, we have treated our SiNWs using 20% HBr before the fabrication of the heterojunction. The other advantages of HBr treatment are reported in our previous work^27^. Amorphous and anatase TiO_2_ thin films can be deposited directly atop the silicon nanowire arrays using spin-coating. To do so, a commercial sol-gel TiO_2_ precursor is purchased from Solaronix. If desired, thermal crystallization from amorphous to anatase TiO_2_ can then be achieved by annealing the samples at 550 °C for 1 hour in a tube furnace. The TiO_2_ layer is spin-coated on a glass substrate for thickness investigation. The thickness of the TiO_2_ layer is in the range of 100–120 nm as revealed by profilometry measurement. Figure 1(c,d) show the top-view and 45° tilted SEM image of the amorphous TiO_2_ layer deposited atop of nanowire arrays. Similarly, Fig. 1(e,f) show the top-view and 45° tilted SEM image of the anatase TiO_2_ layer atop the nanowire arrays. Pristine SiNWs and the amorphous and anatase phases of the TiO_2_ layer on top of SiNWs can be verified using Raman micro-spectroscopy as shown in Fig. 2 which are consistent with the literatures^27^. We have observed four characteristics peaks at 145 cm^−1^, 395 cm^−1^, 518 cm^−1^ and 634 cm^−1^ as illustrated in Fig. 2. We have noticed an intense characteristics peak at 518 cm^−1^ because the SiNWs peak coincides with the anatase TiO_2_ peak at that wavenumber.

We have observed the XRD patterns to confirm the amorphous and anatase phase of the TiO_2_ films. Figure S2 shows the XRD spectra of the as-cast and annealed TiO_2_ deposited on FTO glass substrates. We observed no sharp peak in the XRD pattern for the as-cast, indicating that there is no crystallized phase, and hence it is amorphous TiO_2,_ as shown in Fig. S2 (in black). By annealing the film at 550 °C for 1 hour the appearance of strong peaks for the TiO_2_ proves the presence of anatase phase (in red). These intense peaks are indexed for anatase TiO_2_ phase crystal as (101), (004) and (105) planes respectively. The chemical composition of the amorphous and anatase TiO_2_ are probed with energy dispersive spectroscopy (EDS) as shown in Fig. S3 in the supplementary information section. The EDS spectra reveal no impurities before and after the annealing. The carbon, silicon and copper peaks in the EDS spectra come from the sample holder and contamination inside the EDS chamber. Thus the EDS peaks are similar before and after the annealing process.

**Figure 3:**
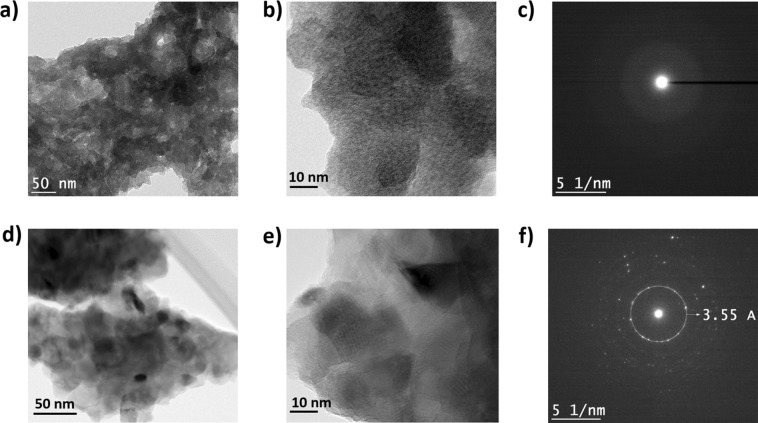
(**a**) TEM micrograph of the amorphous TiO_2_. (**b**) High-resolution TEM images of the same amorphous TiO_2_ showing small crystal grains. (**c**) The corresponding SAED pattern with no observable lattice spacing. (**d**) TEM micrograph of the anatase TiO_2_. (**e**) HRTEM image of the anatase TiO_2_ and (**f**) The corresponding SAED pattern and lattice spacing of the anatase TiO_2_ indexed to (101) phase.

The crystalline structure and phase of the TiO_2_ films are further investigated using transmission electron microscopy (TEM). The TEM images in Fig. 3(a,d) show several crystallites present in the amorphous and the anatase films with bright & dark contrast originating from thickness variation in the films. High-resolution TEM (HRTEM) and selected area electron diffraction (SAED) show the crystalline nature of the films. The HRTEM images are shown in Fig. 3(b,e). The intensity of the diffraction rings indicates that the particle crystallites have a good crystalline nature. We observed no distinctive diffraction rings and lattice fringes for the amorphous phase (Fig. 3(c)). In Fig. 3(f) good diffraction rings and lattice fringes with a d-spacing of 0.355 nm, corresponding to the (101) plane of highly crystalline anatase phase TiO_2_ is observed which is well consistent with XRD results^8,28–30^.

### Materials characterization

The morphology of the SiNWs and TiO_2_ films are characterized using a Hitachi SU 8230 ultra-high-resolution field emission scanning electron microscope. Raman peaks are collected using a WITec alpha 300 micro-Raman system with a 532 nm laser. X-ray powder diffraction (XRD) patterns are collected using a Bruker-AXS D8 Advance X-ray diffractometer with CuKα1 radiation (λ = 1.5406 Å) in the range of 10–60° (2θ) with a step size of 0.02°. Transmission electron microscope (TEM) characterization is carried out using a JEOL 2100 F TEM equipped with an EDX spectrometer and SAED.

### Photodetector device fabrication

**Figure 4:**
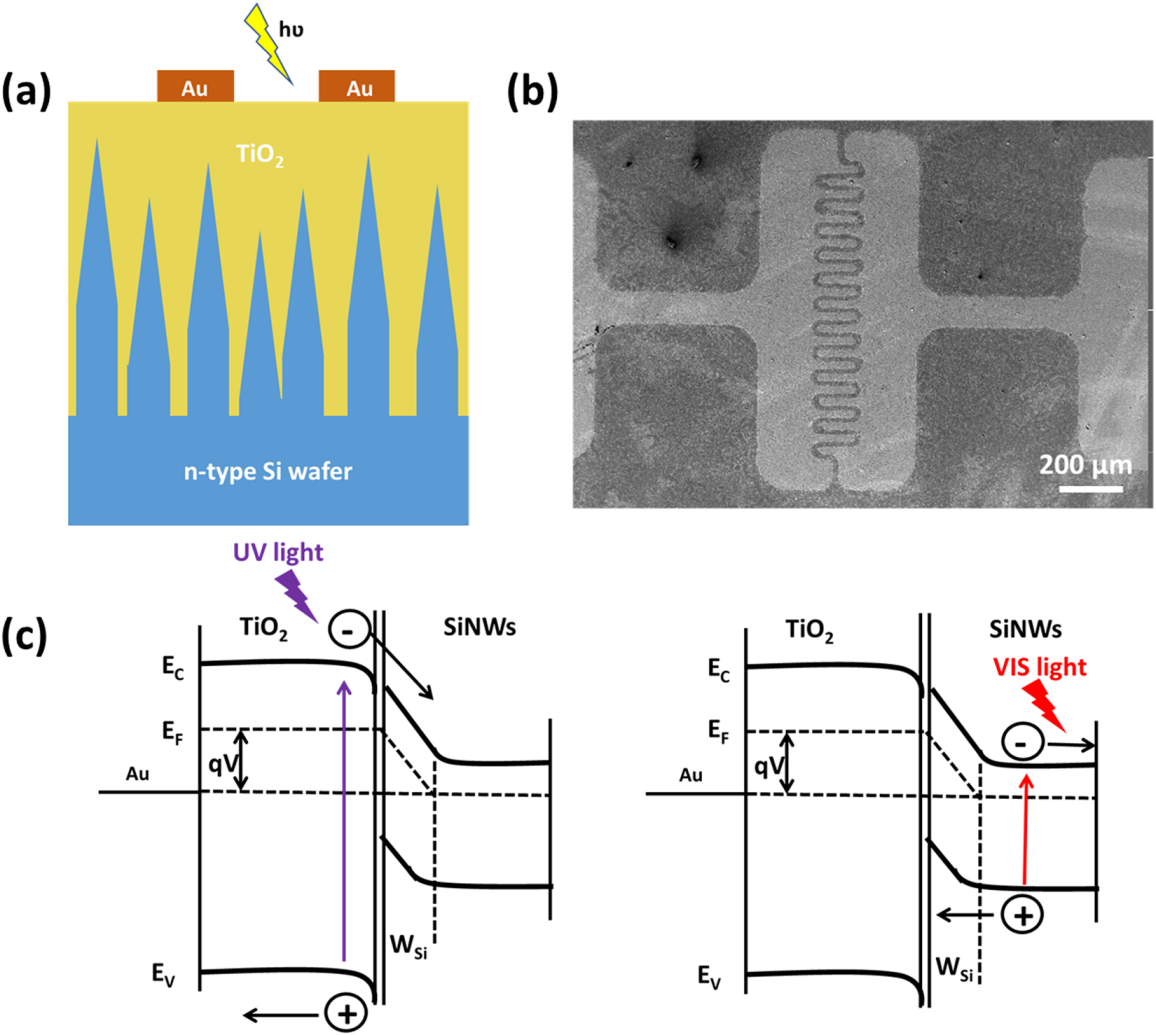
(**a**) Schematic of the photodetector device architecture. (**b**) SEM image of the top view of the final photodetector devices. (**c**) Simplified band-diagram of the device.

Figure 4a shows a schematic of the photodetector device structure. Gold pads with 50 µm channels have been deposited on top of the whole SiNWs/TiO_2_ heterojunction through mask evaporation technique in order to fabricate the photodetectors. For the bare SiNWs control device, gold pads with 50 µm channels are deposited directly on top of the SiNWs. Scanning electron microscope (SEM) image of the top view of the photodetector is depicted in Fig. 4b. Figure 4c shows the simplified band diagram of the heterostructure. Under UV illumination, the photo-generated carriers are produced mostly in the TiO_2_ region due to its larger bandgap. However, visible illumination generates carriers mostly in the silicon. Hence the photodetector has the capacity to detect both UV and VIS light.

### I-V curves, spectral photoresponse, and time-dependent measurements

I-V characteristics curves are measured using a Keithley 2400 source meter under AM1.5 G illumination at 100 mW.cm^−2^ using a Newport solar simulator.The photocurrent spectral measurement is performed with a Xenon lamp attached to a TRIAX320 monochromator, using a chopper and a lock-in amplifier as previously reported^31^. In the setup, the light from the Xenon lamp first passes through the monochromator to perform a 10 nm–step scan from 300 nm to 700 nm. Then the excitation light is modulated at 30 Hz before illumination of the sample, which is biased at 1 V. The photocurrent is measured utilizing a lock-in amplifier. The responsivity is calculated by dividing the photocurrent by the power of the incident light at each wavelength, which is measured with a calibrated photodiode (Newport 918D). Finally, EQE measurements are done with the same setup as reported^32^. The transient photoresponse is probed by illuminating the device using a continuous-wave 532 nm laser chopper-modulated at a frequency of 830 Hz. A mechanical chopper is used to regulate light exposure onto the device. To monitor the photoresponse during the on-off cycles, a change in voltage is obtained by measuring the photovoltage with an Agilent DSO-X 3034 A oscilloscope through a load resistance of 1 GΩ^33^. The rise and decay time constant of the photodetectors is determined using exponential curve fitting.

## Results and Discussions

**Figure 5:**
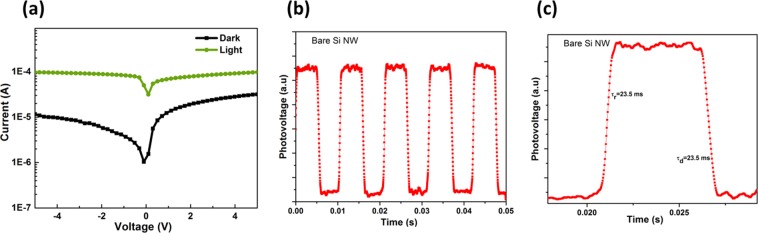
Control silicon-only device. (**a**) Current-voltage device characteristics in the dark and under AM 1.5 G illumination. (**b,c**), Transient photo-response measurements under 532 nm laser illumination.

**Figure 6:**
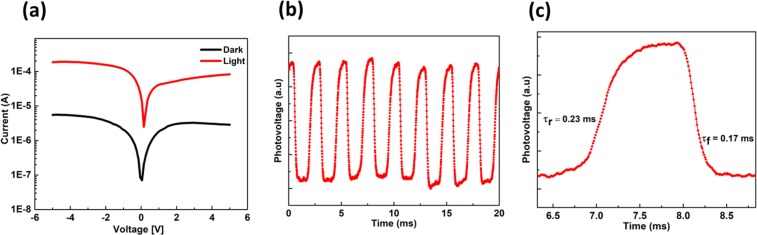
Heterojunction device with the amorphous TiO_2_ layer atop the silicon nanowire arrays. (**a**) Current-voltage device characteristics in the dark and under AM 1.5 G illumination. (**b,c**) Transient photo-response measurements under 532 nm laser illumination.

As a control sample, the logarithmic current-voltage (*I-V*) device characteristic in the dark and under illumination for bare silicon nanowire array samples without any TiO_2_ is shown in Fig. 5(a). We can notice a small response between dark and illumination conditions suggesting a Schottky diode. The measured rise and decay time constants for this bare silicon nanowire device are 23.5 ms, as shown in Fig. 5(b,c). With the amorphous TiO_2_ layer deposited atop of the vertically aligned silicon nanowire arrays, Fig. 6(a) a two- orders of magnitude photo-response is probed. The measured rise/decay times are 0.23 ms/0.17 ms as shown in Fig. 6(b,c). These response time constants are very small compared to previously-published results^12^. A smaller rise/decay time is, of course, critical for a fast response and wide range of applications.

**Figure 7:**
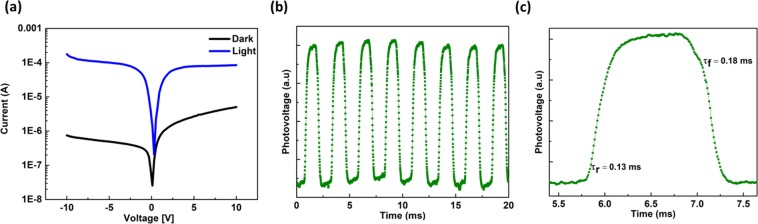
Heterojunction device with the anatase TiO_2_ layer atop the silicon nanowire arrays. (**a**) Current-voltage device characteristics in the dark and under AM 1.5 G illumination. (**b,c**) Transient photo-response measurements under 532 nm laser illumination.

We have also compared their performances against identical heterojunction devices using thermally-converted anatase TiO_2_. Figure 7(a) shows the photocurrent to be is about 2.5 orders of magnitude higher than the dark current. Transient behaviours are shown in Fig. 7(b,c)with rise/decay time at 0.13 ms/0.18 ms.

Compared with bare silicon nanowire devices, the rise/decay times of the SiNWs/amorphous TiO_2_ heterojunction based photodetectors decrease because the heterojunction promotes charge carrier separation (cf. Fig. 4c). However, the amorphous and anatase TiO_2_ based devices are comparable. This could be attributed to the similar structural and electronic properties of amorphous and anatase TiO_2_ as suggested by literature^14^. However, amorphous TiO_2_ presents a disordered arrangement of O and Ti atoms. Thus, anatase devices perform slightly better than amorphous devices.

**Figure 8:**
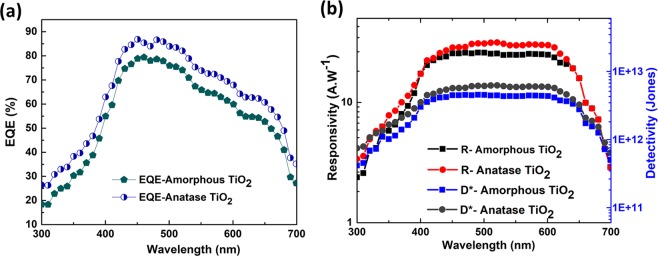
(**a**) EQE of the photodetectors using amorphous and anatase TiO_2_,-based heterojunctions. (**b**) Responsivity and detectivity of the photodetectors using amorphous and anatase TiO_2_,-based heterojunctions.

The external quantum efficiency (EQE) of the silicon-TiO_2_ photodetectors are shown in Fig. 8(a). The EQE of the amorphous TiO_2_ heterojunction-based photodetector reaches up-to 31% in the UV 78% in the visible. In contrast, the EQE of the anatase TiO_2_ heterojunction-based photodetector is slightly higher and reaches up to 38% in the UV and up-to 85% in the visible. Hence the amorphous TiO_2_-based device performs close to the anatase-based detectors. The spectral responsivity shown in Fig. 8(b) is also a crucial parameter to determine the figure-of-merit of a photodetector device. The responsivity for the amorphous TiO_2_-based devices reaches up-to 6.0 A/W in the UV and 25.0 A/W in the visible under a small (1 V) external bias. Meanwhile, the responsivity measured from the anatase TiO_2_-based devices reaches up-to 8 A/W in the UV and 29 A/W in the visible. The high EQE and responsivity values under near-UV and visible illumination show its potential for broadband sensing applications.

We can also calculate directly the detectivity (D*) of our photodetectors as shown in Fig. 8(b). The detectivity (D*) characterizes the capability of a photodetector to detect the weakest light signal^34^. In general, the detectivity can be calculated from the relation^31,33^:

1 $${D}^{\ast }=\frac{\sqrt{A}R}{\sqrt{2q{I}_{d}}}$$

where *A* is the active area, *R* is the responsivity, *q* is the elementary charge and *I*_*d*_ is the dark current. It is obviously important to have very low dark current in order to detect weak signals. From the responsivity and *I-V* measurements, the specific detectivity of our amorphous TiO_2_-based photodetector was then calculated to be 1.05 × 10^12 ^cm Hz^1/2^ W^−1^ or *Jones* at 350 nm and 4.12 × 10^12^ Jones at 600 nm by applying a 1 Volt bias. In contrast, the detectivity for the anatase TiO_2_-based photodetectors is found to be 2.29 × 10^12^ Jones at 350 nm and 8.75 × 10^12^ Jones at 600 nm. Hence our amorphous TiO_2_-based photodetectors show excellent performances which are also comparable to the anatase TiO_2_-based devices.

**Figure 9:**
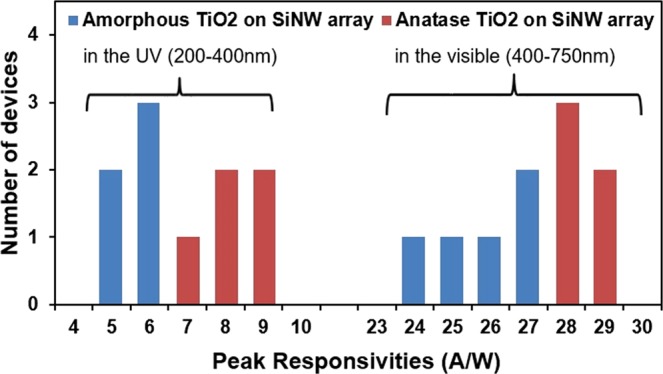
Maximum responsivity values in the UV and the visible region for devices fabricated with amorphous and anatase TiO_2_.

Figure 9 shows the histogram of the statistical variations in peak responsivities in the UV and visible regions for five amorphous TiO_2_ -based photodetectors and five anatase TiO_2_-based devices. All of the amorphous TiO_2_-based photodetectors show peak responsivities between 5–6 A/W in the UV and 24–27 A/W in the visible. In contrast, the anatase TiO_2_-based photodetectors show peak responsivities between 7–9 A/W in the UV and 28–29 A/W in the visible. These results suggest great device performances and good reproducibility. Albeit slightly lower, the performances of the amorphous TiO_2_-based devices remain comparable to the best anatase TiO_2_-based devices.

### Performance comparison against previous reports

Table 1 presents a detailed comparison of the performances of our silicon nanowires - amorphous TiO_2_ heterojunction-based photodetector devices against previous reports on similar devices. We can observe that this work reports the fastest UV/VIS photodetector device with the highest responsivity using a low applied bias. The proposed fabrication method using an all solution-based approach in ambient condition is also the most readily accessible amongst those other alternative methods using only fume-hood chemistry with only a final shadow mask evaporation step. As explained in detail in our previous report, the HBr treatment prior to the heterojunction fabrication leads to the dangling bond passivation at the interface^27^. This explains the outstanding performance of our photodetectors as compared to the literature.

**Table 1:** Comparison of the key parameters of our photodetector with the published data.

Paper	Rise time	Decay time	Responsivity	External bias	Processing temperature	Morphology	Method
Banerjee *et.al*.(this work)	0.23 ms	0.17 ms	6 A/W@ 350 nm 25 A/W@ 600 nm	1 V	Room temperature	n-SiNWs/amorphous TiO_2_ thin film	All solution based
Hosseini *et. al*.^13^	60 s	150 s	N/A	4.2 V	850 °C	p-Si/ n TiO_2_ nanostructures	Thermal oxidation growth
Chang *et. al*.^19^	12 s	30 s	N/A	0–1 V	400 °C	p-Si/ TiO_2_ nanotube	Atomic layer deposition
Selman *et. al*.^20^	50.8 ms	57.8 ms	0.45 A/W @ 325 nm	5 V	550 °C	p-Si/ rutile TiO_2_ nanorod	Sputtering
Yoon *et.al*.^35^	N/A	N/A	N/A	5 V	200 ^o^C	p-Si/ amorphous TiO_2_	Atomic layer deposition
Ji *et. al*.^12^	0.05 s	0.05 s	17 mA/W @365 nm 2 A/W @565 nm	−2 to −4V	600 ^o^C	n-Si/ TiO_2_ nanorod	Sputtering

## Conclusion

We report on the facile fabrication of high-performance photodetectors using a heterojunction formed between a silicon nanowire arrays produced by galvanic displacement etching of a commercial n-type silicon wafer covered with a commercial sol-gel TiO_2_ precursor. As we show, this sol-gel TiO_2_ precursor can be kept in the amorphous phase or thermally crystallized to anatase TiO_2_ to produce photodetectors with a broad response covering the near-UV and the visible regions. While devices using the crystallized anatase TiO_2_ still show slightly better performances, the photodetectors using the amorphous TiO_2_ show excellent peak responsivities at 6 A/W in the near-UV and 25 A/W in the visible. The peak EQE of these photodetector devices reaches 31% in the near-UV detection and 78% in the visible. The fast rise/decay time constants at 0.23 ms/0.17 ms and high specific detectivities (D*) suggest their high-capability to detect minimal signal for a wide range of applications. Overall, these results suggest a tremendous potential for these ultra-low-cost all solution-based heterojunction photodetector devices.

## Supplementary information

Supplementary Information
