# Supplementary material for: Low-cost photodetector architectures fabricated at room-temperature using nano-engineered silicon wafer and sol-gel TiO2 – based heterostructures
Source: Sci Rep. 2019 Nov 29;9:17994. doi: 10.1038/s41598-019-54481-8 (PMC6884441; doi:10.1038/s41598-019-54481-8)
Supplement: Supplementary file 1 — Supplementary Information [file 41598_2019_54481_MOESM1_ESM.pdf]

Supplementary Information

Low-cost photodetector architectures fabricated at room-temperature using nano-engineered silicon wafer and sol-gel TiO_2 – based heterostructures

Debika Banerjee[1a], Ivy M. Asuo[1a,2], Alain Pignolet[2] and Sylvain G. Cloutier[1*]

[1]Dept. of Electrical Engineering, École de Technologie Supérieure, 1100 Notre-Dame Ouest, Montréal, QC, Canada H3C 1K3
[2]Institut National de la Recherche Scientifique (INRS), 1650 Boul. Lionel Boulet, Varennes (QC), J3X 1S2, Canada
[a]Authors contributed equally
** Address correspondence to sylvaing.cloutier@etsmtl.ca.*

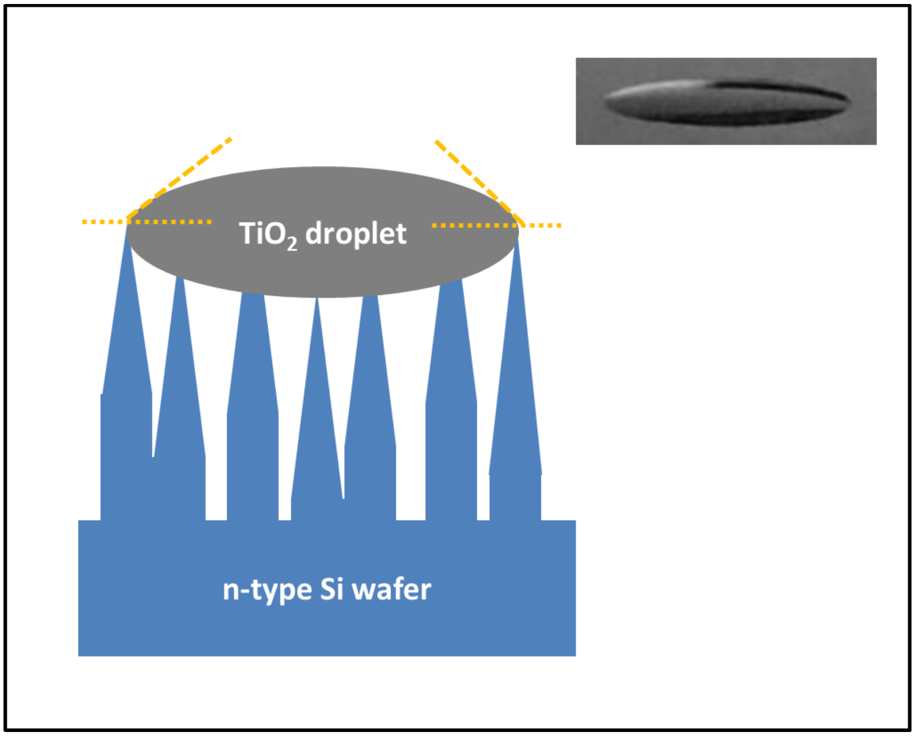

Figure S1: Schematic of the TiO_2 droplet on nanowire surface (grayscale image of the droplet is shown in the inset)

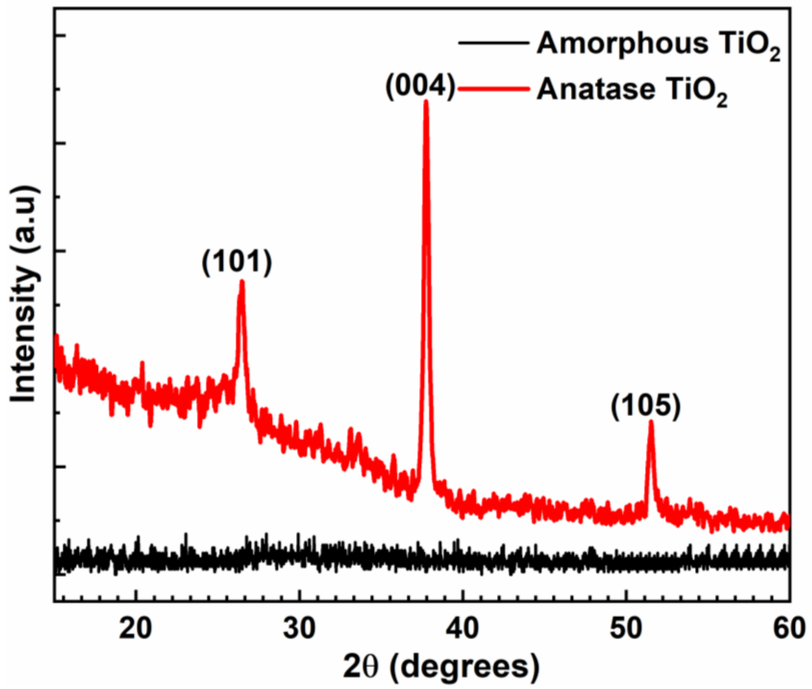

Figure S2: X-ray diffraction pattern of the amorphous (black) and anatase (red) TiO_2 films on FTO substrate.

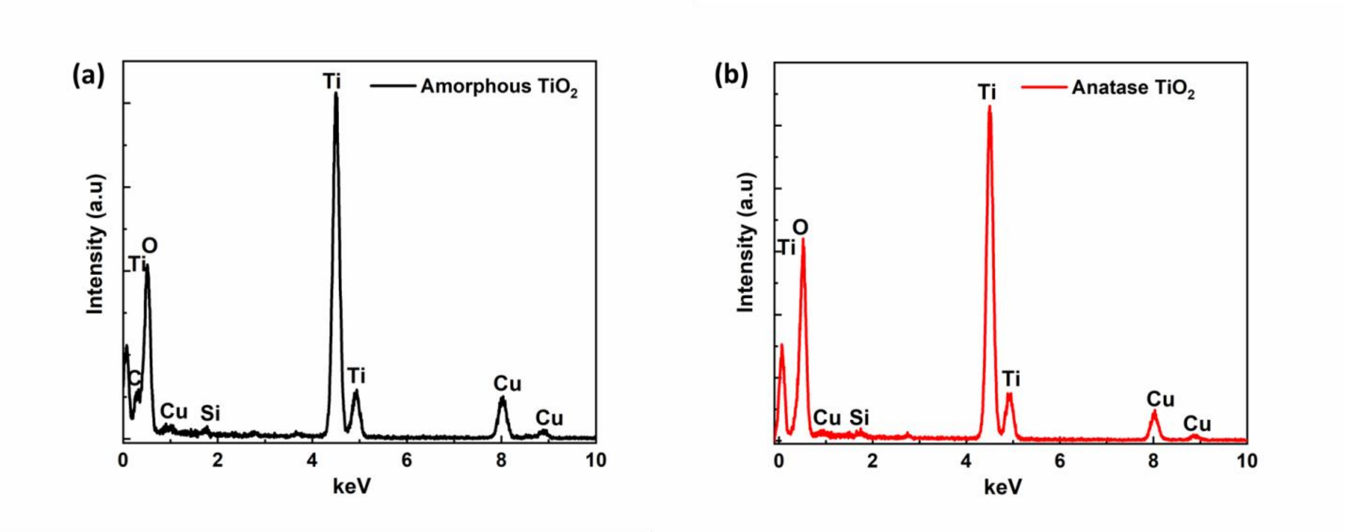

Figure S3: EDS analysis of (a) Amorphous and (b) Anatase TiO_2 samples.
